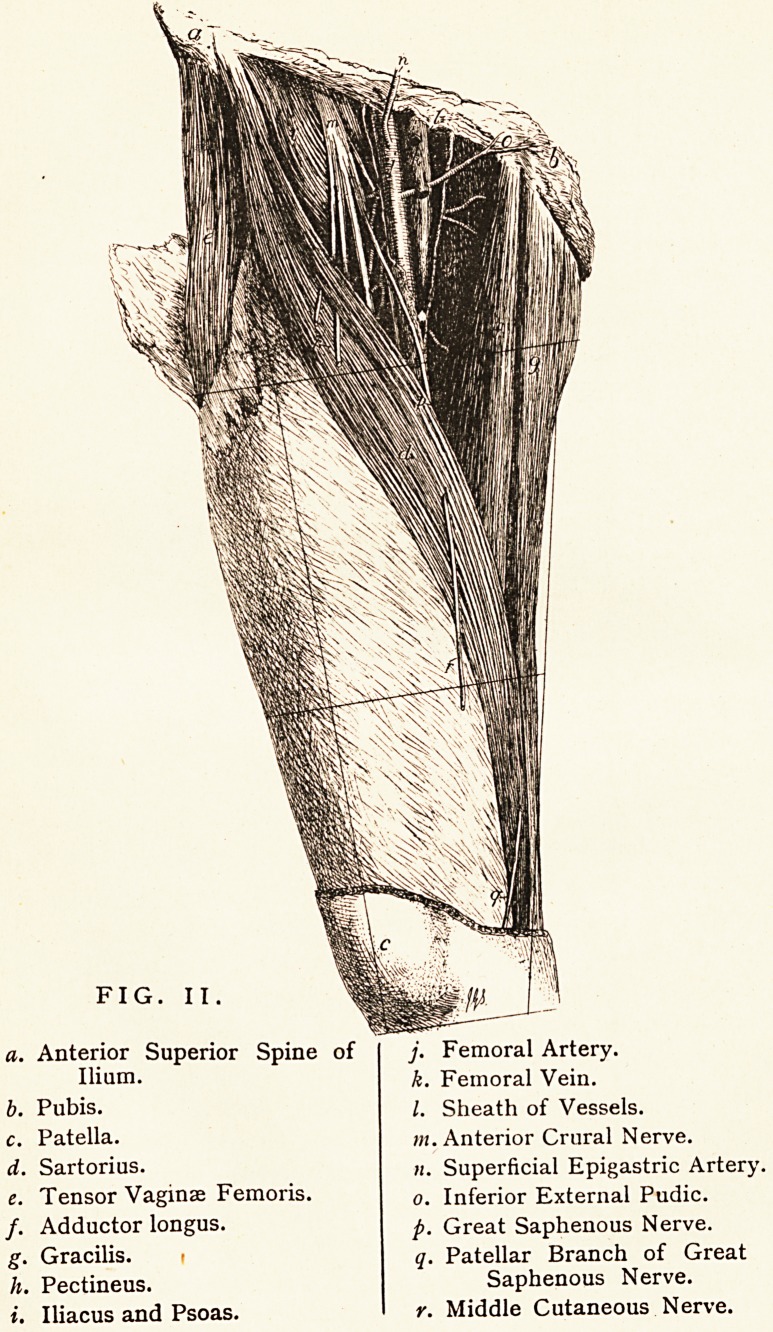# Joseph Griffiths Swayne

**Published:** 1903-09

**Authors:** 


"*Scire est nescire, nisi id me*
*Scire alius sciret.*"

SEPTEMBER, 1903.

### JOSEPH GRIFFITHS SWAYNE, M.D. (Lond.).

SOMETHING more than the ordinary obituary notice is called for by the decease of one who has been quite the *doyen* of the medical profession of the West of England for many years past.

The name of Swayne has been a household word for several decades, and by his death we lose almost the last of a band of medical men who, in the middle of the last century, did much to advance the fame of Bristol as a medical centre. The Bristol Medico-Chirurgical Society and Journal Committee have to acknowledge with gratitude much assistance from him for many years: as an original member rarely absent from a meeting, as President, and as a frequent contributor both to debates and articles in the Journal, he has done as much as was in his power to further the prosperity of the Society. The members will be glad to have the photographic souvenir opposite, representing him as he was known to us about ten years ago. We have to thank Dr. Swayne's family for the photograph and its reproduction.

Joseph Griffiths Swayne was born on October 18th, 1819, at a house in St. James' Barton, on the north side of the passage leading to St. James' Square. He was the second son of Mr. John Champeny Swayne, Senior Consulting Accoucheur to the Bristol Lying-in Institution, Lecturer on Midwifery at the Bristol Medical School, and grandson of the Rev. George Swayne, who was for nearly sixty years Vicar of Pucklechurch, Gloucestershire. His mother was the eldest daughter of Dr. Thomas Griffiths, who at that time lived and practised in a house in St. James' Barton, on the site of which Messrs. Cordeux's establishment now stands. He received his early education at the Bristol College, at that time a flourishing proprietary school situated in Park Row, we believe where the Jewish Synagogue now stands, and at that time presided over by Dr. Jerrard, a pedagogue of considerable local eminence. Here he had as contemporaries several whose names have become well known locally, such as the brothers Prichard, the Rev. S. Wayte, Professor Stokes, Mr. H. Nash, Ralph Bernard, and others. (The Bristol College was, we believe, the first attempt at a school of the character of the present public schools in the neighbourhood).

On leaving school he was apprenticed to his father, and was also entered as a pupil at the Bristol Medical School, and at the Royal Infirmary, as dresser, under Mr. Richard Lowe. Here it may be of interest to note that in those days (1837) students at the Infirmary consisted of three classes—house pupils, dressers (or practically surgeon's apprentices, who served for three years), and "walking pupils" (who were allowed to see the practice); it is also noteworthy that at that time many of the lectures at the Medical School commenced at 7.15 a.m. At the Infirmary at that time the surgeons were Messrs. Richard Smith, Jun., Richard Lowe, Nathaniel Smith, Morgan, and Harrison, and the physicians Drs. J. C. Prichard, George Wallis, John Howell, and Henry Riley. Augustin Prichard was a contemporary student. He soon after entered the then newly-formed University of London, and on passing the intermediate examination for the degree of Bachelor of Medicine, he obtained honours in Anatomy and Physiology, being among the first three in that subject.

After completing or nearly completing his term in Bristol

FIG. I.

*a.* Fascia lata.
*b.* Coccyx.
*c.* Superficial fascia of thigh.
*d.* Deep layer of superficial fascia.
*e.* Gluteus maximus.
*f.* External sphincter ani.
*g.* Levator ani.
*h.* Inferior hemorrhoidal vessels.

FIG. II.

*a.* Anterior Superior Spine of Ilium.
*b.* Pubis.
*c.* Patella.
*d.* Sartorius.
*e.* Tensor Vaginæ Femoris.
*f.* Adductor longus.
*g.* Gracilis.
*h.* Pectineus.
*i.* Iliacus and Psoas.
*j.* Femoral Artery.
*k.* Femoral Vein.
*l.* Sheath of Vessels.
*m.* Anterior Crural Nerve.
*n.* Superficial Epigastric Artery.
*o.* Inferior External Pudic.
*p.* Great Saphenous Nerve.
*q.* Patellar Branch of Great Saphenous Nerve.
*r.* Middle Cutaneous Nerve.

he entered at Guy's Hospital, and obtained the diplomas of M.R.C.S. and L.S.A. in 1841 ; he also studied in Paris, and in 1843 graduated as M.B. in the University of London, gaining the gold medals in Medicine and Obstetric Medicine and first-class honours in Surgery; for the gold medal in Medicine he was declared equal with Sir Alfred Baring Garrod. He graduated M.D. in 1845, and for a few years practised in partnership with his father and Mr. S. H. Swayne his younger brother. For a short time he was Demonstrator and then Lecturer on Anatomy at the Bristol Medical School, and in 1845 succeeded his father as Lecturer on Midwifery, which post he held until 1895.

A fact not generally known is that he was engaged for some time on a Manual of Anatomy, the illustrations for which were etched by himself on copper from his own dissections. The publication, however, was forestalled by Ellis's *Dissections;* and as, owing to the liberality with which it was illustrated, his own work could only have been published at a much higher price, he never completed it. We give specimen illustrations of his etchings. (Fig. 1, Perineum, and Fig. 2, Scarpa's Triangle.)

In 1848 he worked vigorously, during the cholera epidemic which then raged in Bristol, in his endeavour to discover the primary cause of the disease, and described a micro-organism which is claimed by many to have anticipated Koch's discovery of the comma bacillus. Some few years ago Mr. Francis Fowke wrote an article in the *British Medical Journal* reviving this claim; he himself did not claim that the micro-organism he discovered was the primary cause, but noted its constant appearance in the dejecta of true cholera cases, and also in water in places where the epidemic was raging. In the pursuit of this research he spent some time in Bridgwater, on the subsidence of the Bristol outbreak, and contracted the disease himself. Fortunately his attack proved to be not a severe one. The publication of the results of these investigations by his co-worker, Dr. Brittan, led to an acrimonious discussion in local medical circles, in which the College of Physicians eventually took part as arbitrator, and vindicated his claim to the discovery. His only complete published work,

*Obstetric Aphorisms for the use of Students*, was published in 1856, and rapidly attained wide popularity, not only in Great Britain but abroad (it has been translated into two Eastern languages, in addition to European ones), and reached its 10th edition in 1893.

His association with his father led to his adoption of Midwifery as a speciality, and in 1853 he was appointed Physician Accoucheur to the Bristol General Hospital. This, we believe, was the first appointment of the kind made outside London. Unfortunately, as is usually the case with pioneers, his early success was small, added to which his health, undermined by an attack of enteric fever, broke down, and he temporarily lost his eyesight. With a view of regaining his health he decided to travel for a year, and in 1858 took the long sea passage to New Zealand, from which he returned a year later completely restored, and to find himself at the commencement of a long career of success. He was singularly fortunate in practice; we have heard him say that he had never lost a patient from septic infection since his student days, when it was the custom for dissection and practical midwifery to be carried on together. This he used to attribute to the fact that he was always very careful about washing his hands. We have also often heard him state that in his opinion those practising midwifery and surgery should avoid wearing either their beards or hair long, as he had noticed that septic poisoning seemed commoner in the practice of those who adopted these fashions than in that of the clean shaven. From these opinions it will be seen that when the application of antiseptics to midwifery was put on a scientific basis, he, although then an old man, was not likely to oppose this as a fad and a novelty, but at once adopted the principle and carried it out with the greatest thoroughness. Apart from the foregoing as regards new methods, he was absolutely without bias in favour of old methods simply because they were old, and was always ready to examine and try any new method or means which might be suggested to him.

At the same time he was a staunch upholder of methods in which there was good; for example, educated as he was in the days of indiscriminate venesection, he never ceased his advocacy of its efficiency in puerperal eclampsia, and lived to see venesection again a recognised method of treatment in this affection.

As a lecturer he was clear, concise, and scholarly, always presenting his subject in a way that showed that he realised the difficulties of the student, while impressing his important points on the memory by illustrations drawn from practical experience of cases under his own observation.

He was a Fellow and Past Vice-President of the Obstetrical Society of London, a Fellow of the British Gynæcological Society, and also of the Gynæcological Society of Boston, U.S.A. In 1894 he presided over the section of Obstetrics at the Meeting of the British Medical Association which was held in Bristol.

In 1895 he resigned his post as Professor of Midwifery in University College, to which he was appointed on the amalgamation of University College and the Bristol Medical School, and was appointed Emeritus Professor. As a mark of their esteem and appreciation of his services, his private colleagues, friends, and pupils entertained him at dinner in February, 1895, and presented him with a handsome service of plate, together with an address.

In his private life he was a man of most abstemious habits, of simplicity of mind and singleness of character. To the first undoubtedly his powerful constitution and prolonged physical powers were no doubt due. From the time of his recovery from his attack of enteric to the day of his death he had never spent a day or even breakfasted in bed. He was an exceptionally early riser; until a few years before his death it was his habit to rise at about 5 a.m., and he followed the practice of taking a cold bath every morning until he was nearly eighty years of age. In his youth he was a great athlete, a good boxer, a great walker, a powerful swimmer, a good horseman, a good oar, and no mean rifle shot. He kept up gymnastic exercises, of which he was very fond, until he was well over sixty. He was an enthusiastic volunteer until he was unable to find the time to attend drill. While a member of the Bristol Rifles he gained a gold medal presented to the best judge of distance in the battalion. His uncle commanded the Dyrham Company of

Gloucestershire Volunteers in 1804. He was an artist of no mean order, a pupil of S. Jackson, who had considerable local celebrity in the thirties; he gained several prizes at local exhibitions, and was also a good judge of works of art.

He was also possessed of considerable musical ability and a good baritone voice, and was a member of the Cathedral Choral and Bristol Choral Societies. He used often to describe the glee parties held at Richard Smith's house in his student days, when under the presidency of that venerable surgeon the students used to meet and pass the evening in glee-singing and conviviality. Many amusing stories could he relate of the doings of the medical student of his day. He often used to say that Albert Smith's *Medical Student* was a very fair representation of the proceedings of the more lively spirits; in fact, his stories of the "grinders" classes of his student days showed that Albert Smith, in that case at least, did not exaggerate.

His descriptions of the old political elections and of the doings of the public on those occasions, as well as his recollections of the reform riots in Bristol, were most interesting.

He was a strong Conservative in politics, and a high churchman; for many years he was a member of the choir of All Saints, Clifton, and a strong supporter of that church.

With the work of this Journal Dr. Swayne was in hearty sympathy, and he often helped it by original contributions and by review. Although he had attained the highest point of his professional eminence, and although he was brought into contact in his general work with men who were not born when he began practice, he was ever ready to fall in with their often-expressed views of the way in which his contributions were ultimately to appear in print. His reviews of books were most thorough, and he could always give ample reason for any adverse criticisms which he thought it necessary to make.

Few men were more regular in their attendance at the Meetings of the local Medical Societies than Dr. Swayne, and there were not many subjects, even outside his special work, on which he was not able to say something that was of interest or of practical utility. Many of us will never forget the striking way in which he would often intervene in a discussion with some very apposite observation when it seemed that he had not been taking any interest in the question before the meeting.

Readers of this Journal had an opportunity of seeing some evidence of Dr. Swayne's skill in portraiture when in the number for December, 1892,[1] were reproduced some sketches which he made in 1840 in his note-book when he was a student at the School.

It can seldom have occurred in the history of medicine that any member of their staff could have had a longer connection with a hospital and medical school than had Dr. Swayne, extending as it did in one case, either as Physician Accoucheur or Consulting Physician Accoucheur, to a period of exactly fifty years, and in the case of the Medical School to fifty-eight years, for the first fifty years of which time he was engaged in lecturing on Midwifery, and during the last eight years as Emeritus Professor in the same subject, being not only the first Professor of Midwifery but also the first Emeritus Professor ever elected on any subject at Bristol University College. Dr. Swayne was also the first Physician Accoucheur ever elected at the Bristol General Hospital, his election dating from the year 1853, when the Hospital was opened. His position on the staff was not quite that which obtains to-day, as his work was chiefly confined to out-patients, and no beds in the Hospital were set apart for his special department, it being understood that he performed no abdominal operation. Abdominal operations were during Dr. Swayne's period of active work at the Hospital not common, and cases requiring operation were handed over to the surgeon; notwithstanding this, his opinion was always sought and much valued by his colleagues in all abdominal cases. Dr. Swayne was elected Lecturer on Midwifery in the Bristol Medical School in the year 1845, and his connection with the School continued up to the time of his death. He saw the School pass through many trying times and much adversity until finally it rested in its present quarters as a part of Bristol University College. Dr. Swayne took a large share in bringing about this desirable object. Not only did he devote much time to

[1] Vol. x., pp. 286 to 291.

his lectures from which many generations of students profited, and by his general conciliatory spirit smoothed over many difficulties in the working of the School; he continued to the end of his life to take a warm interest in the School, and when the new wing was opened in 1895, he was one of the largest if not actually the largest contributor in money to the funds.

Of Dr. Swayne's personal characteristics as friend, colleague and doctor it would be impossible to speak too highly. In all the relations of life he was all that could be desired—a scholar and a gentleman in the best sense, a most loyal and amiable colleague; by nature he was simple and kindly, the least aggressive of men, and essentially a man of peace; he never appeared to be striving after fame or popularity or working for effect, but strong in the quiet consciousness of power in his own professional sphere, he had no necessity for self assertion, his abilities being recognised and appreciated by all his professional brethren who during his long life were brought into contact with him.

There are few men among us in this neighbourhood who have been engaged in midwifery who have not at one time or another sought his valuable assistance in cases of difficulty; he enjoyed in a remarkable degree the confidence and esteem of all who knew him.

He died painlessly and suddenly in his eighty-third year, "full of days, riches and honour."

---

### BIBLIOGRAPHY OF THE WORK OF JOSEPH GRIFFITHS SWAYNE.

"Introductory Lecture on Midwifery," *Prov. M. & S. J.*, 1846, 497, 511.

"An Account of Certain Organic Cells Peculiar to the Evacuations of Cholera," *Lancet*, 1849, ii. 368, 398, 410.

"On the Varieties of Cranial Presentation," *Prov. M. & S. J.*, 1852, 88, 128, 153.

"Brow Presentation as a Cause of Difficult Labour," *Assoc. M. J.*, 1853, 299.

"Case of Fracture of the Cranium in an Infant at Birth," *Ibid.*, 901.

"Case of Difficult Labour from Hydrocephalus," *Ibid.*, 1854, 921.

"On Embryotomy in Presentations of the Superior Extremities," *Ibid.*, 1855, 917, 930.

*Obstetric Aphorisms*, 1856.

"Case of Rupture of the Uterus during Pregnancy," *Assoc. M. J.*, 1856, 859, 887.

"On Flexions of the Uterus," *Brit. M. J.*, 1857, 456.

"Case Successfully Treated by the Ready Method," *Lancet*, 1857, i. 331.

"The late Case of Manslaughter at Vauxhall," *Ibid.*, 565.

"On Ulceration of the Os Uteri," *Brit. M. J.*, 1858, 430.

"On Supporting the Perinæum," *Ibid.*, 1860, 196.

"Fibrous Tumour of the Uterus," *Ibid.*, 1860, 358.

"Case of Double Monstrosity," *Tr. Obst. Soc. Lond.* (1860), 1861, ii. 320.

"Case of Arm-Presentation with Exomphalos," *Brit. M. J.*, 1861, i. 333.

"Presidential Address to the Bath and Bristol Branch of the British Medical Association," *Ibid.*, 1861, ii. 382, 513, 594.

*Obstetric Aphorisms*, 2nd Ed., 1861.

"Case of Cyanosis," *Brit. M. J.*, 1862, i. 253.

"Statistics of Forceps Delivery," *Ibid.*, 1862, ii. 245.

"Discolouration of the Skin of the Fore-arms and Hands during Pregnancy," *Tr. Obst. Soc. Lond.* (1862), 1863, iv. 18.

*Obstetric Aphorisms*, 3rd Ed., 1863.

"Cases of Puerperal Convulsions," *Brit. M. J.*, 1863, i. 346, 467.

"The Address in Midwifery delivered at the Thirty-first Annual Meeting of the British Medical Association held at Bristol, 1863," *Ibid.*, 1863, ii. 178.

"Case of Cæsarean Section," *Tr. Obst. Soc. Lond.* (1863), 1864, v. 84.

"Cases of Polypus Uteri," *Brit. M. J.*, 1866, i. 69.

"Case of Double Monstrosity," *Tr. Obst. Soc. Lond.* (1866), 1867, viii. 1.

"Changes in the Shape of the Fœtal Head produced by Labour," *Brit. M. J.*, 1867, i. 768.

*Obstetric Aphorisms*, 4th Ed., 1867.

"On the Treatment of Puerperal Convulsions," *Brit. M. J.*, 1868, ii. 151, 420.

"On the Use of Obstetric Instruments," *Ibid.*, 1869, i. 72, 487.

"Case of Puerperal Convulsions," *Ibid.*, 1871, i. 191.

"Treatment of Hæmorrhage Arising from Retention of the Secundines after Abortion," *Ibid.*, 1871, ii. 201.

"Hydrate of Chloral in Puerperal Convulsions," *Ibid.*, 752.

*Obstetric Aphorisms*, 5th Ed., 1871.

"Rupture of the Membranes Before the Completion of Pregnancy," *Brit. M. J.*, 1872, i. 184.

"Perinæal Lacerations," *Ibid.*, 1872, ii. 93.

"Case of Puerperal Convulsions," *Ibid.*, 1873, i. 668.

"On the Induction of Premature Labour," *Ibid.*, 1874, ii. 165, 479, 555, 707.

"Remarks on the Adhesion of the Placenta," *Ibid.*, 1875, i. 801.

"Obstetrical Statistics," *Ibid.*, 1875, ii. 232, 635.

"The Use of Perchloride of Iron in *Post-partum* Hæmorrhage," *Ibid.*, 522.

*Obstetric Aphorisms*, 6th Ed., 1875.

"On a New Form of Blunt Hook and Sling for Assisting Delivery in Cases of Breech Presentation," *Tr. Obst. Soc. Lond.*, (1875) 1876, xvii. 313.

"On the Relative Frequency of the Different Cranial Positions," *Obst. J. Gr. Brit.*, 1875-76, iii. 372.

"The Use of Forceps in the First Stage of Labour," *Brit. M. J.*, 1877, i. 508.

"Turpentine in *Post-partum* Hæmorrhage," *Ibid.*, 1878, i. 295.

"Case of Puerperal Convulsions," *Obst. J. Gr. Brit.*, 1878–79, vi. 73.
"Remarks on the Effects of Forceps Delivery on the Infant," *Brit. M. J.*, 1878, ii. 459.
"Are First Labours more Dangerous than Others?" *Obst. J. Gr. Brit.*, 1879-80, vii. 65.
*Obstetric Aphorisms*, 7th Ed., 1880.
"On the Treatment of Lacerations of the Cervix Uteri," *Obst. J. Gr. Brit.*, 1880, viii. 705.
"On a New Form of Stem Pessary," *Tr. Obst. Soc. Lond.*, (1882) 1883, xxiv. 220.
"Gangrene during Pregnancy," *Lancet*, 1883, ii. 690.
"A Record of Twenty-one Cases of Placenta Previa," *Bristol M.-Chir. J.*, 1883, i. 166.
"Gangrene of the Thigh during the Seventh Month of Pregnancy," *Tr. Obst. Soc. Lond.*, (1883) 1884, xxv. 215.
*Obstetric Aphorisms*, 8th Ed., 1884.
"Ought Craniotomy to be Abolished?" *Bristol M.-Chir. J.*, 1887, v. 1.
"Cases of Ruptured Uterus," *Tr. Obst. Soc. Lond.*, (1886) 1887, xxviii. 213.
"Hydrocephalus as a Complication of Labour," *Ibid.*, (1887) 1888, xxix. 405.
"The Hour of Delivery," *Bristol M.-Chir. J.*, 1888, vi. 174.
*Obstetric Aphorisms*, 9th Ed., 1888.
"Accidental Hæmorrhage," *Bristol M.-Chir. J.*, 1889, vii. 170.
"Case of Epithilioma of the Body of the Uterus," *Ibid.*, 1890, viii. 119.
"Puerperal Eclampsia," *Ibid.*, 1891, ix. 1.
"Forceps Delivery during the Last Fifty Years," *Ibid.*, 1892, x. 153.
*Obstetric Aphorisms*, 10th Ed., 1893.
"Compression of the Umbilical Cord during Forceps Delivery," *Bristol M.-Chir. J.*, 1893, xi. 229.
"Ergot of Rye as an Oxytoxic," *Ibid.*, 1894, xii. 225.
"On the Treatment of Puerperal Eclampsia occuring during Pregnancy and Presenting no Signs of Labour," *Brit. M. J.*, 1896, i. 523.
"On Occipital Presentations," *Bristol M.-Chir. J.*, 1898, xvi. 108.

## Figures and Tables

**Figure f1:**
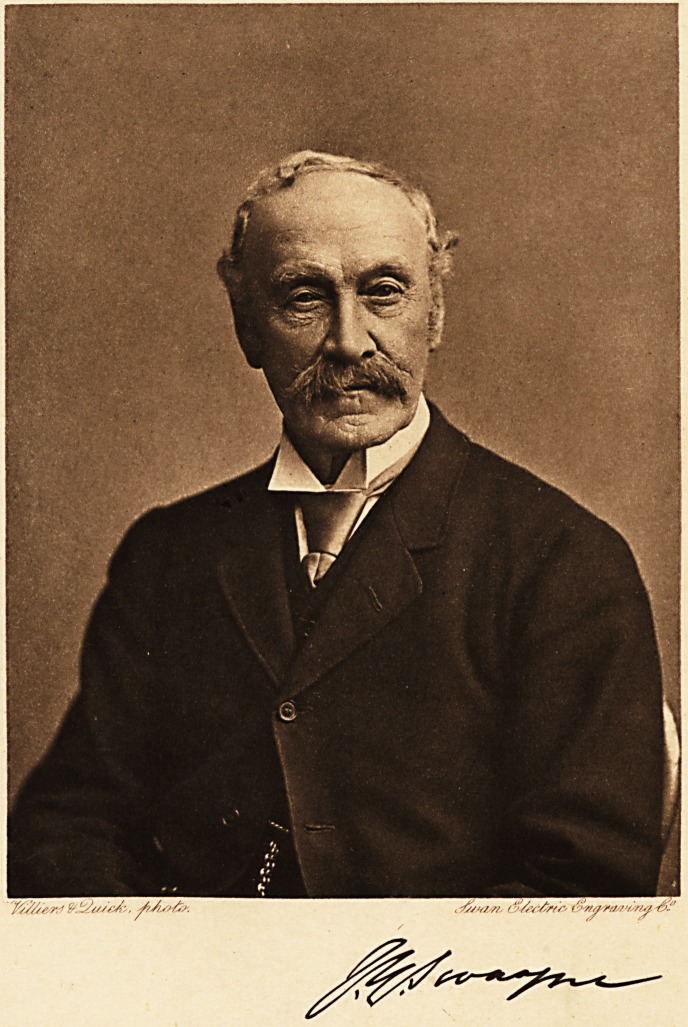


**FIG. I. f2:**
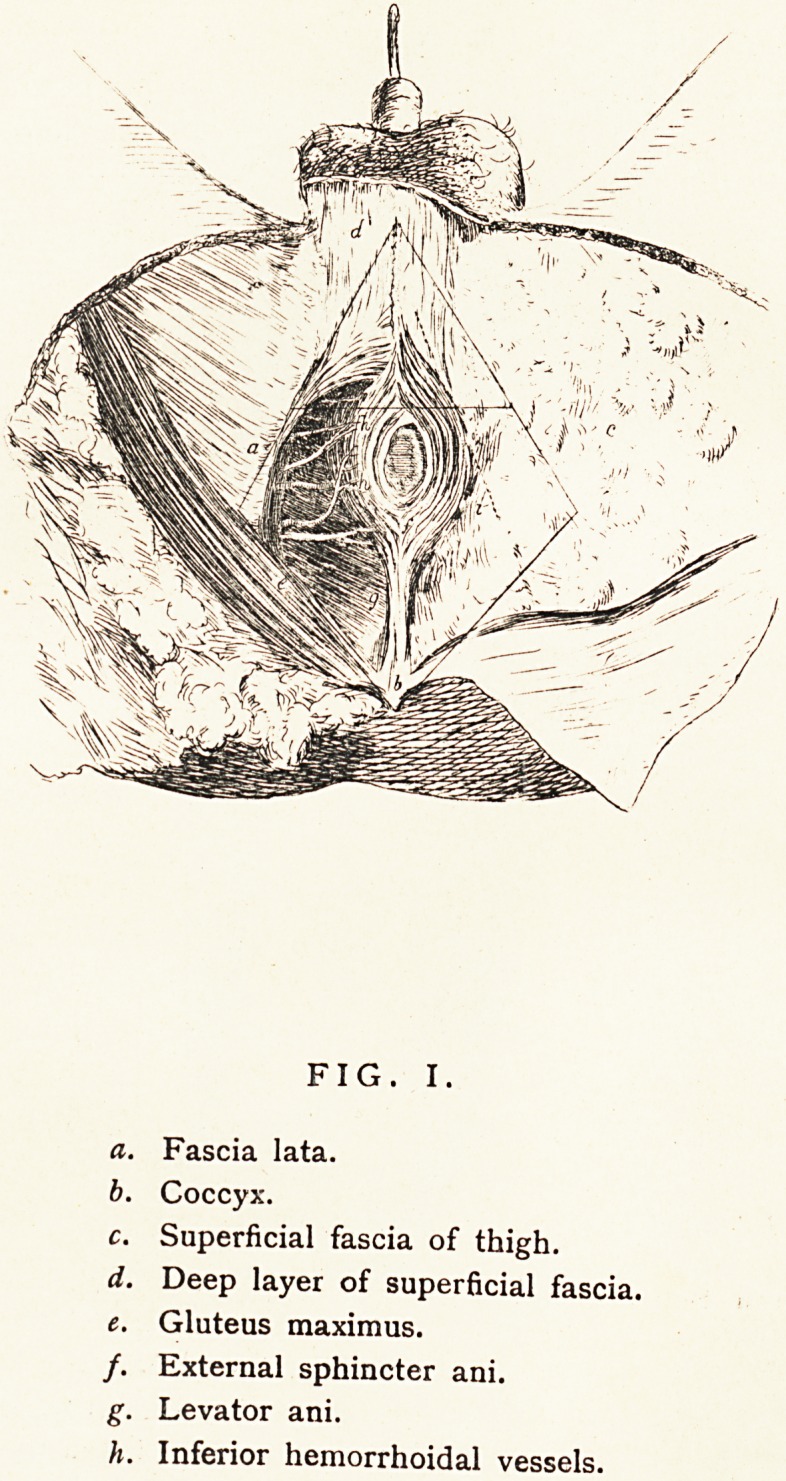


**FIG. II. f3:**